# Use of artificial intelligence in paediatric anaesthesia: a systematic review

**DOI:** 10.1016/j.bjao.2023.100125

**Published:** 2023-02-07

**Authors:** Ryan Antel, Ella Sahlas, Genevieve Gore, Pablo Ingelmo

**Affiliations:** 1 Faculty of Medicine and Health Sciences, McGill University, Montreal, Quebec, Canada; 2 Schulich Library of Physical Sciences, Life Sciences, and Engineering, McGill University, Montreal, Quebec, Canada; 3 Department of Anesthesia, Montreal Children's Hospital, McGill University, Montreal, Quebec, Canada

**Keywords:** artificial intelligence, critical care, machine learning, paediatric anaesthesia, perioperative medicine

## Abstract

**Objectives:**

Although the development of artificial intelligence (AI) technologies in medicine has been significant, their application to paediatric anaesthesia is not well characterised. As the paediatric operating room is a data-rich environment that requires critical clinical decision-making, this systematic review aims to characterise the current use of AI in paediatric anaesthesia and to identify barriers to the successful integration of such technologies.

**Methods:**

This review was registered with PROSPERO (CRD42022304610), the international registry for systematic reviews. The search strategy was prepared by a librarian and run in five electronic databases (Embase, Medline, Central, Scopus, and Web of Science). Collected articles were screened by two reviewers. Included studies described the use of AI for paediatric anaesthesia (<18 yr old) within the perioperative setting.

**Results:**

From 3313 records identified in the initial search, 40 were included in this review. Identified applications of AI were described for patient risk factor prediction (24 studies; 60%), anaesthetic depth estimation (2; 5%), anaesthetic medication/technique decision guidance (2; 5%), intubation assistance (1; 2.5%), airway device selection (3; 7.5%), physiological variable monitoring (6; 15%), and operating room scheduling (2; 5%). Multiple domains of AI were discussed including machine learning, computer vision, fuzzy logic, and natural language processing.

**Conclusion:**

There is an emerging literature regarding applications of AI for paediatric anaesthesia, and their clinical integration holds potential for ultimately improving patient outcomes. However, multiple barriers to their clinical integration remain including a lack of high-quality input data, lack of external validation/evaluation, and unclear generalisability to diverse settings.

**Systematic review protocol:**

CRD42022304610 (PROSPERO).

The use of artificial intelligence (AI) within medicine has seen remarkable growth in recent years, and research regarding future applications remains a rapidly evolving area of study. AI can be broadly defined as the study of algorithms that give machines the ability to reason and perform functions such as problem-solving, object and word recognition, inference of world states, and decision-making.^1^ In practice, this often refers to computer systems that simulate intelligent behaviour such as learning, reasoning, and problem solving.^2^ Recently, the availability of large data collections in combination with these intelligent computing systems have accelerated advancements in the development of AI applications. AI is not a single technology, but rather a range of processes and behaviours generated by computational models and algorithms.^3^ This heterogeneous group of computing systems includes techniques such as machine learning, computer vision, fuzzy logic, natural language processing, advanced robotics, and artificial voice technology.^3^ Machine learning allows computing programmes to learn from and react to data without explicit programming, using either a supervised, unsupervised, or reinforcement learning approach.^4^ In order to achieve this, techniques such as neural networks, decision trees, and Bayesian methods are often used.^4^ Computer vision refers to a computing system's ability to understand images, video, and other visual data such as CT.^5^ As such, computer vision interprets the visual world in numerical or symbolic form to allow for subsequent action.^4^ Fuzzy logic is a superset of conventional (Boolean) logic that incorporates the concept in partial truth, to allow more accurate representations of the real world when performing logic-based tasks.^6^ Meanwhile, natural language processing uses computational techniques to learn, understand, and produce human language content.^7^

The ability of AI to support evidence-based clinical decision-making has led to the rapid development of novel clinical applications in multiple healthcare domains.^3^ The paediatric operating room, and other acute care settings, requires critical and complex decision-making that must be made under stringent time constraints and often is embedded within much uncertainty.^8^ Such settings are also data-rich environments with numerous continuously monitored physiological variables that are responsive to interventions over short periods of time.^9^^,^^10^ As such, critical care settings including the operating room and ICU offer opportunity for applications of AI to enhance the decisions of clinicians, identify and potentially address modifiable patient risk factors, contribute to shared decision-making with patients and their families, prioritise care appropriately, and ultimately enhance automation.^8^ The study of such technologies within adult anaesthesia and pain medicine has been significant, with recent work showing its potential to impact the practice of anaesthesiology ranging from perioperative support to critical care delivery to outpatient pain management.^4^^,^^11^ However, research regarding the use of AI for paediatric anaesthesia has lagged behind.^8^^,^^12^^,^^13^ As such, this systematic review aims to characterise the current application of AI in paediatric anaesthesia and to discuss barriers to the successful clinical integration of such technologies.

## Methods

The Preferred Reporting Items for Systematic Reviews and Meta-Analyses (PRISMA) guidelines for systematic reviews was followed for this review.^14^ This systematic review has been registered with PROSPERO (CRD42022304610), the international registry for systematic reviews.^19^

### Identifying relevant studies

A senior medical librarian searched the following databases from inception until May 2022: Embase (Ovid), Medline (Ovid), Central (Cochrane Library), Scopus, and Web of Science (SCI-EXPANDED, CPCI–S, ESCI). The search strategy used variations in text words found in the title, abstract, or keyword fields, and relevant subject headings to retrieve articles looking at the use of AI for anaesthesia within the paediatric population. Various forms of the central terms ‘artificial intelligence’, ‘pediatric,’ and ‘anesthesia’ were used to identify relevant articles. The search strategy had no language restriction. See Supplementary Appendix A for the full search strategies.

### Study selection

**Fig 1:**
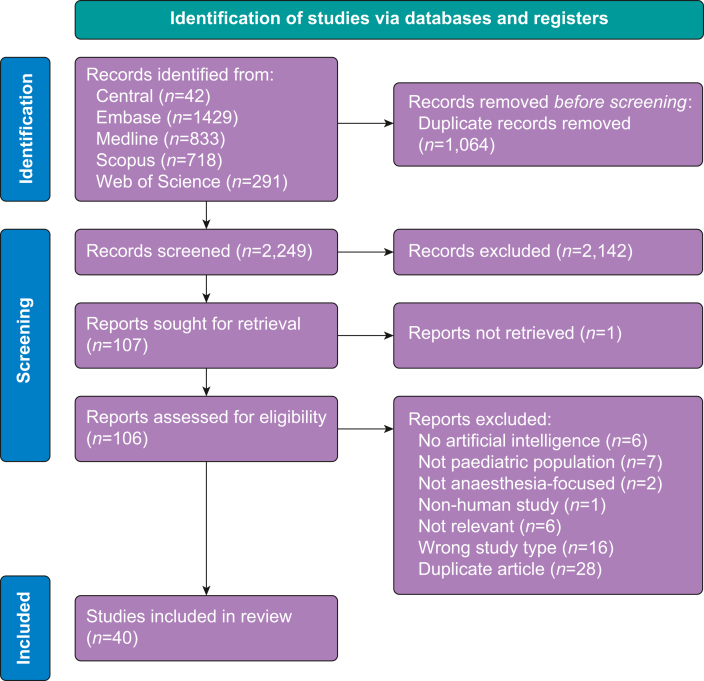
PRISMA flow diagram. PRISMA, Preferred Reporting Items for Systematic Reviews and Meta-Analyses.

All titles and abstracts obtained in the literature search were manually and independently screened by two authors using Rayyan, an online screening tool.^15^ Identified relevant articles then underwent full-text screening independently by two authors, with disagreements resolved through discussion. Articles included in the final review described applications of AI for paediatric anaesthesia. Although there remains disagreement in the literature about whether linear and logistic regression models should be considered a basic form of machine learning (and therefore AI), we have opted to include such models in our review to err on the side of inclusion as was done in comparable studies.^4^^,^^11^^,^^16^^,^^17^ As well, given significant heterogeneity in participant age reporting practices in the collected literature, we opted to include all studies with a reported mean participant age less than 18 yr. Articles written in languages other than English and French, and articles in the form of review articles, conference abstracts, editorials, and commentaries were excluded. There was no further limitation on study design. A PRISMA diagram was used to record the screening decisions (Fig 1).^14^

### Data extraction

After the selection of studies, data from each article were extracted and organised into 16 categories in a standardised data extraction form developed in Microsoft Excel (Microsoft Corp., Redmond, WA, USA). This was done independently by two authors to record the information and synthesise it in summary format. Extracted information included author name(s), year of publication, title, location of study, study design, goal of study, target study population, mean study population age, number of included participants, description of discussed AI intervention, domain of AI used, data source, evaluations of AI tool accuracy/efficacy, main results of the study, identified barriers to clinical integration of described application, and a category for additional pertinent information of interest.

### Risk of bias assessment, collating, summarising, and reporting results

Information in the data extraction form was collated, and the findings and trends as they relate to AI in paediatric anaesthesia were recorded and summarised. The risk of bias of included publications was assessed depending on publication type using previously published risk assessment tools such as the Template for Intervention Description and Replication (TIDieR) checklist and guide for reporting of interventions,^18^ and the Prediction Model Study Risk of Bias Assessment Tool (PROBAST) for reporting of prediction models.^20^

## Results

### Study characteristics

The characteristics of the included studies are described in Table 1 and the results of the current literature search are shown in the PRISMA diagram (Fig 1). From an original search including 3313 references, 2249 articles were screened after duplicates were removed, and 107 were selected for full-text review. This resulted in 40 articles being included in this review. Studies included in this review were published between 1994 and 2021. Included publications were from 17 different countries, with most from the USA (13; 32.5%) and Canada (10; 25%). Studies ranged in size from 5 participants^42^ to 186 492 participants.^31^ Although most studies (30; 75%) described applications for use within a specific paediatric population (such as patients undergoing a specified type of surgical procedure, or children with a certain medical condition), fewer studies (10; 25%) more generally described applications for use with paediatric patients undergoing surgery/anaesthesia without further specification. A large number of studies included in this review used data obtained from medical records (17; 42.5%) as the basis of their AI application, whereas fewer used data compiled during surgical procedures such as recorded patient physiological data (8; 20%). Data gathered from nursing reports (1; 2.5%), questionnaires/scales completed by patients, physicians, or both (2; 5%), hospital-wide curated databases (2; 5%) or other registers of clinical data (9; 22.5%) were discussed as well. One study did not report the source of their data (1; 2.5%).

**Table 1:** Characteristics of included publications. *n*, number of patients included in study.

Study	Description of intervention	Target population	Data source
Agostoni, 2011	Model to assess the predictability of age against dependent variables of complications during sedation for endoscopy	Patients under light sedation for endoscopy (*n*=457)	Curated database from single hospital
Al-Alawi, 2022	Prediction system for effect of propofol and isoflurane on peripheral venous pressure waveforms	Pyloric stenosis and craniosynostosis patients (*n*=48)	Collected data from surgical procedures
Alassaf, 2019	Predictive model to identify risk factors for blood transfusion requirement in children with developmental dysplasia of the hip	Patients who underwent anterior open reduction and/or acetabular osteotomy with and without femoral shortening (*n*=524)	Electronic medical records
Ali, 2020	Predictive model to identify risk factors for postoperative thrombotic complications	Children who underwent surgery with cardiopulmonary bypass (*n*=369)	Electronic medical records
Ammer, 2021	Predictive model to identify disease-specific risk factors for the composite binary endpoint ‘anaesthesia-related complications’	Patients diagnosed with mucopolysaccharidoses who had at least one procedure under anaesthesia (*n*=99)	Electronic medical records
Ansermino, 2009	Software tool with algorithm monitors the physiological data to guide clinician actions during anaesthesia	Surgical patients undergoing routine anaesthesia (*n*=19)	Collected data from surgical procedures
Ariza, 2014	Predictive model to determine the current prevalence of serious and non-serious adverse events for children who required anaesthesia care at a general gastroendoscopic service	Children younger than 12 yr who underwent a gastrointestinal endoscopic procedure (*n*=1742)	Clinical charts and nurse registers
Bassanezi, 2013	Predictive model to evaluate risk of postoperative vomiting in paediatric oncologic patients	Children with a diagnosis of malignancy undergoing surgery (*n*=188)	Hospital data
Cheon, 2016	Predictive model to identify the incidence and predictors of unplanned postoperative intubation in paediatric patients	Patients who underwent surgery (*n*=87 920)	National Surgical Quality Improvement Program (NSQIP-P) database
Chiesa, 2021	Predictive model to allow clinicians to predict the need for sedation during radiation therapy	Patients with an oncological diagnosis for which radiation treatment had been prescribed (*n*=99)	Data collected during clinical appointments
Chini, 2019	Algorithm that distinguishes non-anaesthetised from deeply anaesthetised states and predicts anaesthetic concentration as a proxy for anaesthetic depth	Neonates and infants who were scheduled for an elective surgical procedure (*n*=35)	Electronic medical records and from the in-house Anaesthesia Information Management System
Cho, 2015	Predictive model to determine the relationship between tracheal tube sizes and ultrasonographic tracheal diameter	Children scheduled to undergo ambulatory ophthalmic surgery requiring general anaesthesia (*n*=126)	Not stated
Dosani, 2009	Algorithms for tracking dynamic physiological monitoring for use during anaesthesia to run context-sensitive monitoring	Paediatric surgical patients (*n*=38)	Data collected in real-time during anaesthesia
Fairley, 2019	Algorithm to optimise scheduling and sequence operating room procedures to minimise delays caused by PACU unavailability using procedure and recovery duration	Paediatric surgical patients (*n*=18 015)	Electronic medical records
Fishman, 2021	Model to determine if polysomnographic cardiorespiratory outcomes could predict the presence of postoperative adverse respiratory events	Children with confirmed neuromuscular disease undergoing surgical intervention under general anaesthesia (*n*=61)	Electronic medical records
Galvez, 2017	Algorithms to distinguish patients that have invasive ventilation with either a laryngeal mask airway or a tracheal tube from those that have noninvasive ventilation during surgery	Patients undergoing myringotomy, tonsillectomy, adenoidectomy or inguinal hernia repair procedures (*n*=900)	Electronic anaesthesia records
Guevara, 2017	Predictive model for postoperative blood loss and mortality	Children underwent corrective cardiac surgery to repair tetralogy of Fallot (*n*=60)	Electronic medical records
Hancerliogullari, 2017	Model of multi-criteria analysis to assess the relative importance of criteria used for selecting anaesthesia method for circumcision surgery	Patients undergoing circumcision (study population size not stated)	Questionnaires completed by expert paediatric surgeons
Hino, 2017	Prediction model for the incidence of emergence agitation	Children scheduled to undergo general anaesthesia (*n*=120)	Collected data from surgical procedures and postoperative care unit
Hu, 2021	Predictive model to help clinicians understand the risk factors for neonatal postoperative mortality	Neonates undergoing surgery (*n*=481)	Data collected by anaesthetic clinical information system
Jalali, 2020	Algorithms to track the behaviour of dynamic physiological systems to enable context-sensitive monitoring of six physiological variables	Paediatric surgical patients (*n*=8)	Recorded physiologic data during surgical procedures
Jiao, 2020	Model to predict surgical case durations	Paediatric patients undergoing general, cardiothoracic, orthopaedic, otolaryngology, ophthalmology, plastic, urology, gynaecology, transplant, neurosurgery, gastroenterology, haematology/oncology, dentistry, and pain medicine procedures (*n*=53 783)	Electronic medical records
Kawaguchi, 2015	Predictive model to identify patients at high risk for failed early extubation in the operating room	Patients who underwent Fontan procedure for underlying cardiac disease and extubated in the operating room (*n*=88)	Paper based charts and provincial internet-based patient information sharing system
Khan, 2021	Model to perform EEG-based anaesthetic depth monitoring	Paediatric surgical patients (*n*=60)	Clinical data set of EEG headset recordings
Kim, 2021	Predictive model for estimating gastric fluid volume using ultrasonography in infants	Infants who were scheduled to undergo general anaesthesia with tracheal intubation (*n*=192)	Data collected from ultrasound assessments
Lee, 2018	Prediction model for tracheal tube depth using neck CT images	Patients who had undergone neck CT imaging (*n*=499)	Electronic medical records
Lin, 2021	Predictive model to identify risk factors for postoperative delirium	Patients who had major elective surgery (*n*=6691)	Data collected via nursing reports
Matava, 2020	Algorithm to assist intubation via real-time during video laryngoscopy or bronchoscopy (draw box on videos around vocal cords and trachea)	Patients undergoing bronchoscopy or videolaryngoscopy (*n*=395)	Clinical dataset of video laryngoscopy and bronchoscopy videos
Nafiu, 2019	Predictive model to identify children at risk for postoperative care unit intravenous opioid requirement	Children scheduled for painful ambulatory surgical procedures (*n*=1134)	Pain scales performed in postoperative care unit and demographic surveys
Nasr, 2020	Predictive model of perioperative morbidity in children	Children undergoing noncardiac surgical procedures (*n*=16 724)	Electronic medical records
Packiasabapathy, 2021	Predictive model to determine the association of quantitative pupillometry measures with postoperative respiratory depression	Children scheduled for elective outpatient adenotonsillectomy for obstructive sleep apnoea or recurrent adenotonsillitis (*n*=220)	Pupil response measurement dynamics collected pre-, intra-, and postoperatively
Robles-Rubio, 2020	Model to rapidly classify breathing patterns (pause, movement, synchronous breathing, asynchronous breathing, or unknown in signals recorded from dual belt respiratory inductance plethysmography	Infants in the surgical recovery room (*n*=21)	Collected data in the immediate postoperative period
Safranek, 2022	Clinical dashboard visualising variation in paediatric opioid administration to enable physicians to track their practice and ultimately address unnecessary variation in clinical practice	Patients undergoing outpatient surgeries (*n*=24 332)	Electronic medical records
Shim, 2021	Prediction model for optimal tracheal tube depth in paediatric patients	Patients received postoperative ventilation (*n*=834)	Electronic medical records and postoperative chest radiographs
Smith, 1994	Application for detecting cardiogenic oscillations in capnographs and removing their effect from the final results for capnogram analysis	Patients monitored via capnography (*n*=5)	Capnography data collected from patients in hospital
Spencer, 2015	A model to assess the relationship between the sonographically measured antral cross-sectional area and endoscopically suctioned gastric volumes	Fasted patients presenting for upper gastrointestinal endoscopy (*n*=100)	Perioperative ultrasound data
Tao, 2021	Predictive model of risk factors for perioperative respiratory adverse events	Children who underwent general anaesthesia along with elective surgery with tracheal intubation (*n*=476)	Collected clinical data
Vlasov, 2021	Model to assess different types of anaesthesia in the surgical correction of congenital malformations in children and identify the association of risk factors for death in selected methods of anaesthesia	Newborns and infants with congenital malformations who received phased surgical treatment (*n*=150)	Collected clinical data
Ward, 2021	Algorithm to predict adolescents at risk of prolonged opioid use after surgery and to identify factors associated with this risk	Patients who underwent a surgical procedure under general anaesthesia (*n*=186 492)	Medical claims data from a national insurance provider database
Zhang, 2022	Predictive model to identify associated risk factors of perioperative respiratory adverse events	Children undergoing elective airway surgery under general anaesthesia (*n*=709)	Demographic surveys and medical records

### AI domains used

Multiple domains of AI were used within the applications described in the literature, such as machine learning, fuzzy logic, natural language processing and computer vision (Table 2). Machine learning was the most represented with 38 included studies describing its use,^13^,^23^,^28^,^29^,^31^,^32^^,^^34^, ^35^, ^36^^,^^38^, ^39^, ^40^^,^^42^,^44^,^46^,^47^^,^^49^, ^50^, ^51^^,^^53^, ^54^, ^55^, ^56^, ^57^, ^58^, ^59^, ^60^, ^61^, ^62^, ^63^, ^64^, ^65^, ^66^, ^67^, ^68^^,^^70^ whereas two applications incorporated the use of fuzzy logic^21^^,^^22^ and only one study described the use of each of natural language processing^23^ and computer vision.^13^ Many branches of machine learning were discussed, including regression models,^29^,^45^,^46^,^49^,^50^,^51^^,^^55^, ^56^, ^57^, ^58^, ^59^, ^60^, ^61^, ^62^, ^63^, ^64^, ^65^, ^66^, ^67^, ^68^ decision trees,^23^^,^^33^, ^34^, ^35^ expert systems,^59^ K-means classifiers,^35^ K-nearest neighbours,^29^^,^^44^ Bayesian approaches,^29^ neural networks,^23^^,^^25^^,^^34^^,^^45^ random forest models,^23^,^29^,^31^^,^^45^, ^46^, ^47^ and support vector machines.^34^^,^^45^^,^^70^ Regression models were the most commonly described, with one study describing a linear regression model,^50^ 23 studies describing logistic regression models,^29^,^36^,^46^^,^^50^, ^51^, ^52^, ^53^, ^54^, ^55^, ^56^, ^57^, ^58^, ^59^, ^60^, ^61^, ^62^, ^63^, ^64^, ^65^, ^66^, ^67^, ^68^ two studies describing least absolute shrinkage and selection operator (LASSO) regression models,^31^^,^^49^ and one study describing an elastic net regression model.^45^ Only one study described the use of computer vision, which performed single-shot image analysis using a neural network.^13^ Natural language processing was also only described in one study to extract data from medical records.^23^ Of note, some studies described the use of multiple branches of machine learning, either in combination or as means of comparison, to find the most suitable technique to use within their desired application. For instance, Hu and colleagues^29^ compared the use of logistic regression, boosting, Bayes approaches, K-nearest neighbours, and random forest models to find the most effective technique applicable to risk factor identification for postoperative mortality in neonates. See Table 2 for an overview of discussed AI techniques within the collected literature.

**Table 2:** Branches of artificial intelligence described for use within paediatric anaesthesia.

Branch of AI	Description	Studies using such branch
Fuzzy logic	A superset of conventional (Boolean) logic that has been extended to handle the concept of partial truth – truth values between ‘completely true’ and ‘completely false.’^6^	Hancerliogullari and colleagues^21^ Bassanezi and colleagues^22^
Natural language processing	Computational techniques to learn, understand, and produce human language content.^7^	Jiao and colleagues^23^
Computer vision	Family of applications to interpret and understand the visual world by extracting useful information from digital images, most often developed with machine learning techniques.^24^	Matava and colleagues^25^
Machine learning	Software algorithms to identify patterns in very large datasets.^26^	
Bagged tree	Manipulation of training data by generating a large number of pseudo datasets by resampling the original observations with replacement to reduce variance, resulting in an ensemble of decision trees which are averaged to make the best overall prediction.^27^	Khan and colleagues^28^
Bayes	Probabilistic classification methods based on Bayes' theorem with the assumption of independence between features using training datasets to make predictions.^11^	Hu and colleagues^29^
Boosting: extreme gradient boosting; gradient boosting	Sequentially uses multiple weak classifiers to augment each other by assigning weights to the outputs obtained. Correct classifications from the first decision are given a higher weight and inputted to the next decision. After numerous cycles, the boosting method combines these weak rules into a single powerful prediction rule.^30^	Ward and colleagues^31^ Jiao and colleagues^23^ Fairley and colleagues^32^ Hu and colleagues^29^
Decision trees	Classifies data items by posing a series of questions about features associated with the items to split the dataset into respective classes. Each split has an edge that connects either to a new decision node that contains another feature to further split the data into homogenous groups or to a terminal node that predicts the class.^33^	Galvez and colleagues^34^ Robles-Rubio and colleagues^35^ Jiao and colleagues^23^ Cho and colleagues^36^
Dynamic linear model	Method for time series data analysis and short-term forecasting.^37^	Ansermino and colleagues^38^ Dosani and colleagues^39^ Jalali and colleagues^40^
Expert system	System containing a knowledge base and inference/rules engine – a set of rules for applying the knowledge base to situations provided to the programme. This is used for make a logical prediction about events taking place in the future or reach a logical conclusion about why an event occurred in the past.^41^	Smith and colleagues^42^
K-means classifier	Divides a number of data points into a number of clusters based on the nearest mean.^11^	Robles-Rubio and colleagues^35^
K-nearest neighbours	A non-parametric, supervised learning classifier, which uses proximity to make classifications or predictions about the grouping of an individual data point.^43^	Al-Alawi and colleagues^44^ Hu and colleagues^29^
Neural networks	Network of nodes that communicate with other nodes via connections. Connections between nodes are weighted based upon their ability to provide a desired outcome, becoming strengthened when their neurones have correlated outputs.^33^	Galvez and colleagues^34^ Jiao and colleagues^23^ Shim and colleagues^45^ Matava and colleagues^13^
Random forest	An extension of decision trees that produces multiple decision trees using a subsample of features to create each decision tree. Trees then predict an outcome, and the majority vote among trees is Used as the model's final class prediction.^33^	Shim and colleagues^45^ Jiao and colleagues^23^ Chiesa and colleagues^46^ Safranek and colleagues^47^ Hu and colleagues^29^ Ward and colleagues^31^
Regression: Linear, Logistic, Elastic Net, LASSO	Characterising the strength of the relationship between a dependent variable and one or more explanatory variables.^48^	Shim and colleagues^45^ Zhang and colleagues^49^ Guevara and colleagues^50^ Agostoni and colleagues^51^ Alassaf and Reitsma^52^ Ali and colleagues^53^ Ammer and colleagues^54^ Ariza and colleagues^55^ Cheon and colleagues^56^ Chiesa and colleagues^46^ Fishman and colleagues^57^ Hino and colleagues^58^ Hu and colleagues^29^ Kawaguchi and colleagues^59^ Kim and colleagues^60^ Lee and colleagues^61^ Lin and colleagues^62^ Nafiu and colleagues^63^ Nasr and colleagues^64^ Packiasabapathy and colleagues^65^ Spencer and colleagues^66^ Tao and colleagues^67^ Vlasov^68^ Ward and colleagues^31^
Support vector machines	Classifies data by creating a decision boundary, known as the hyperplane, that is orientated as far as possible from the closest data points from each observed class of data.^69^	Galvez and colleagues^34^ Shim and colleagues^45^ Chini and colleagues^70^

### Analysis of described AI applications

**Fig 2:**
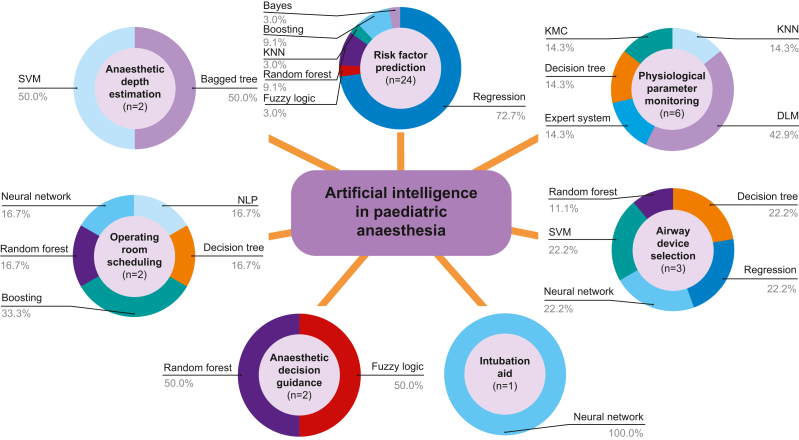
Mapping of artificial intelligence applications in paediatric anaesthesia by application type. Number of studies describing applications within each category are indicated in parentheses. Percentages represent the proportion of applications within such application category using specified artificial intelligence branch. DLM, dynamic linear model; KMC, K-means classifier; KNN, K-nearest classifier; NLP, natural language processing; SVM, support vector machine.

**Fig 3:**
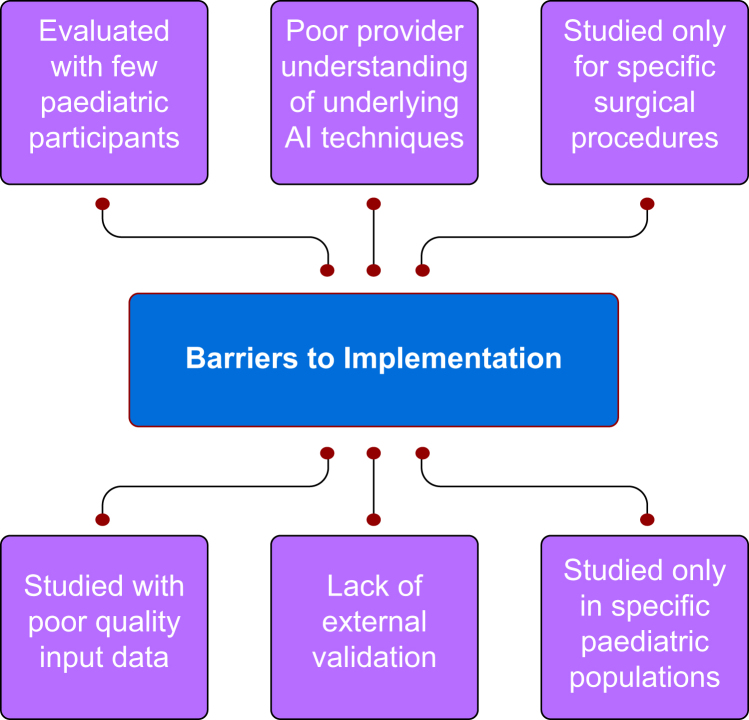
Identified barriers to the clinical implementation of described artificial intelligence applications within paediatric anaesthesia.

Although included studies were heterogenous in terms of the intended use of their described application within paediatric anaesthesia, we were able to characterise interventions into seven categories for use within the perioperative and intraoperative setting (Fig 2): (1) risk factor prediction tools, (2) anaesthetic depth estimation models, (3) operating room scheduling aids, (4) anaesthetic medication/technique decision guidance tools, (5) intubation assistance tools, (6) airway device selection tools and (7) physiological variable monitoring tools. Identified barriers to the successful clinical implementation of such tools included applications developed using data from only specific surgical procedures and limited patient populations, tools developed using small population sizes and incomplete input data, and a lack of external validation, feasibility testing, or evaluation of the clinical efficacy of described applications. A summary of such barriers is included in Fig 3.

### Risk factor prediction tools

Applications using AI for risk factor prediction were described in 24 studies.^22^,^29^,^31^,^46^^,^^49^, ^50^, ^51^, ^52^, ^53^, ^54^, ^55^, ^56^, ^57^, ^58^, ^59^, ^60^, ^61^, ^62^, ^63^, ^64^, ^65^, ^66^, ^67^, ^68^ In most of these described models, machine learning was used to identify factors that allow clinicians to assess the risk of unwanted perioperative events in given patients. For instance, Ammer and colleagues^54^ used logistic regression to identify risk factors associated with anaesthesia-related complications in children with mucopolysaccharidoses based upon patient data including medical comorbidities and previous treatments received. Similarly, Nafiu and colleagues^63^ used logistic regression to determine factors associated with increased recovery room intravenous opiate requirements after surgery. Eleven of these studies based their risk factor identification upon data obtained from medical records,^29^,^49^,^50^^,^^52^, ^53^, ^54^, ^55^^,^^57^,^59^,^61^,^64^ whereas the rest used data from hospital databases^22^^,^^51^ or other collections of clinical information.^31^^,^^46^^,^^56^^,^^58^^,^^60^^,^^62^^,^^63^^,^^65^, ^66^, ^67^, ^68^

### Anaesthetic depth estimation models

Two included studies described using AI to estimate anaesthetic depth in patients undergoing anaesthesia.^28^^,^^70^ Both of these applications relied upon analysis of real-time EEG data using machine-learning techniques (support vector machine and bagged tree classifier, respectively).^28^^,^^70^

### Operating room logistics

Studies by Fairley and colleagues^32^ and Jiao and colleagues^23^ discussed the use of AI to optimise operating room and PACU scheduling. Fairley and colleagues^32^ accomplished this by using machine learning (gradient boosting) to estimate the required PACU time for different types of surgical procedures in order to minimise PACU occupancy. Meanwhile, Jiao and colleagues^23^ used multiple machine-learning techniques (decision trees, neural networks, and a random forest model) to estimate surgical case durations to optimise operating room scheduling. Furthermore, Jiao and colleagues^23^ also used natural language processing as a means to extract relevant data from collected patient medical records.

### Anaesthetic medication/technique decision guidance tools

Decision-support tools were described by Hancerliogullari and colleagues^21^ and Safranek and colleagues^47^ to help clinicians make appropriate choices regarding anaesthetic medications/techniques for given patients. Hancerliogullari and colleagues^21^ described a model based upon fuzzy logic to represent the relative importance of the criteria used for selecting anaesthesia methods for circumcision surgery. Data were obtained from questionnaires distributed to expert clinicians. Safranek and colleagues^47^ developed a clinical dashboard using machine learning with data from medical records to visualise opioid administration practices. This was meant to allow physicians to track and address unnecessary variation in opioid administration to patients undergoing surgery.

### Intubation assistance tools

Only one included study described an application to help providers with tracheal intubation in real time.^13^ Matava and colleagues^13^ developed a computer vision-based application to identify normal vocal cords and tracheal airway anatomy during an intubation. Their tool was based upon video analysis using a neural network and was trained on a clinical dataset of laryngoscopy and bronchoscopy videos.

### Airway device optimisation tools

Three included studies described the use of AI to guide clinicians in selecting and using appropriate airway devices for a given patient.^34^^,^^36^,^45^ Cho and colleagues^36^ developed a model to determine the relationship between tracheal tube sizes and ultrasonographic tracheal diameter using decision trees and regression analysis. Galvez and colleagues^34^ compared decision trees, a neural network and a support vector machine to find the most accurate method to distinguish patients that have invasive ventilation with either a laryngeal mask airway or a tracheal tube from those that have noninvasive ventilation during surgery. This was done in anticipation of future work to develop a tool that aids clinicians in the insertion and removal of appropriate invasive airway devices. Shim and colleagues compared multiple branches of machine learning (neural network, random forest model, elastic net regression model, and a support vector machine) to develop a model that can predict optimal tracheal tube depth in paediatric patients.^45^

### Physiological variable monitoring tool

Applications intended to monitor physiological variables during anaesthesia were described in six studies.^35^^,^^38^, ^39^, ^40^^,^^42^,^44^ Al-Alawi and colleagues^44^ used a K-nearest neighbour model to monitor changes in peripheral venous pressure waveforms in relation to propofol and isoflurane administration. Ansermino and colleagues,^38^ Dosani and colleagues,^39^ and Jalali and colleagues^40^ described applications to monitor physiological signals during procedures in order to give early warning of potential upcoming adverse events. Variables such as heart rate, end-tidal carbon dioxide concentration, minute ventilation, and ventilatory frequency were monitored. All three studies described the use of a dynamic linear model for their application. Smith and colleagues^42^ discussed an expert system to detect cardiogenic oscillation in capnographs in order to adjust for their affect when performing automated capnogram analysis. Robles-Rubio and colleagues^35^ developed a model to classify breathing patterns from signals recorded with dual belt respiratory plethysmography using decision trees and a K-means classifier.

### Risk of bias assessment

**Fig 4:**
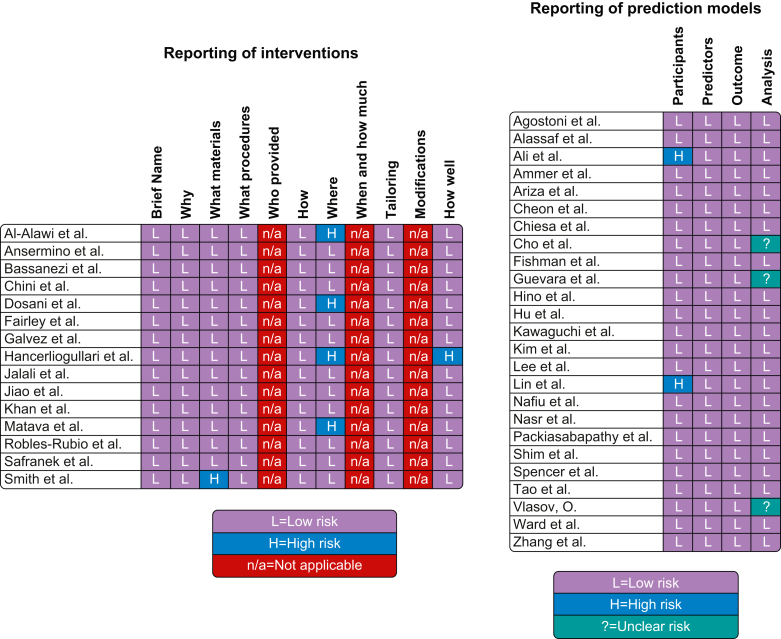
Risk of bias assessment of included studies using the TIDieR checklist^18^ for reporting of interventions (left) and the PROBAST checklist^20^ for reporting of prediction models (right). PROBAST, Prediction Model Study Risk of Bias Assessment Tool; TIDieR, Template for Intervention Description and Replication.

The risk of bias assessment of included studies is summarised in Fig 4. Most included articles were seen to have a low risk of bias. However, the main methodological limitation observed across studies was poor reporting of intended settings of application use (such as the patient population for which a given tool was designed, or the surgical setting for which a tool was intended).

## Discussion

AI is becoming increasingly integrated into medical practice, and its use in anaesthesia has been gaining interest.^4^ As is the case with many other emerging areas of research, paediatrics has not been a primary focus of AI studies to date.^12^ A recent review of the use of AI within adult anaesthesia by Hashimoto and colleagues^11^ described six themes of AI applications from 173 included studies: (1) depth of anaesthesia monitoring, (2) control of anaesthesia, (3) event and risk prediction, (4) ultrasound guidance, (5) pain management, and (6) operating room logistics. However, current research regarding the potential application of AI in paediatric anaesthesia remain less well characterised despite many of the above themes being likely transferrable to a paediatric setting. To address this, the current systematic review provides an overview of the literature describing applications incorporating AI for use within paediatric anaesthesia. Broadly speaking, research regarding the use of AI within paediatric anaesthesia has the ultimate goal of assisting clinicians in providing safe and efficient patient care. A large variety of tools were described to accomplish this, with many different branches of AI discussed. Described applications focused on intraoperative patient monitoring, guiding clinicians in making choices regarding anaesthetic management, and patient risk factor identification. Tools used to aid operating room scheduling logistics were also described. AI domains including machine learning, fuzzy logic, natural language processing, and computer vision were applied. Machine learning was seen to be the most widely applied domain of AI, with regression analysis being the most commonly used technique. This is in line with previously published work, which suggests that statistic-based approaches such as logistic regression remain the most commonly used technique for outcome prediction within anaesthesia.^8^ Although this may be simply attributable to the greater comfort many clinicians have with using and interpreting such well-known statistical methods, a recent review article supports their widespread use by arguing that there is no performance benefit of more sophisticated machine-learning methods over logistic regression for many clinical prediction models.^71^

Although the described AI applications have the potential to contribute to patient care, multiple barriers to their current implantation are recognised in the current literature. Many of these barriers are not unique to the application of AI within paediatric anaesthesia, as a recent viewpoint highlighted four major barriers to the broader application of big data systems in healthcare: (1) model calibration, validation, and updating; (2) data quality and data heterogeneity; (3) user trust in such systems; and (4) difficulty selecting which decisions to support and how to communicate this information.^8^^,^^72^

Most of the studies included in this review were performed only using data from specific surgical procedures and limited patient populations, thus making the generalisability of the described application uncertain.^23^,^31^,^32^,^35^,^40^,^45^,^49^,^51^,^58^^,^^60^, ^61^, ^62^^,^^66^,^70^ Similarly, most applications were developed using small population sizes and incomplete input data (missing/unreliable information), which limited the ability of the studies to accurately evaluate the performance of the developed tools.^29^,^44^,^45^,^50^,^56^,^59^,^64^ As is becoming increasingly evident, challenges regarding the incorporation of AI systems in medicine translate far beyond the immediate AI application and extend throughout the organisation of our healthcare systems.^73^ For such technologies to reach their full potential, emphasis needs to be placed upon establishing, storing, cleaning, and sharing accurate data collections between groups, institutions, and organisations.^74^ While ensuring that databases are sufficiently large is important, it is vital that such databases are of good quality. Ultimately, collaborative data systems that provide complete and meaningful information gathered from all members of the healthcare continuum will be needed to optimise such technologies.^74^ However, this will undoubtedly require commitment, raised awareness, funding, and widespread infrastructure development. Furthermore, the need for the development of methods to consistently monitor and audit such data collections will be needed.

Many tools described in this review lacked formal external validation, feasibility testing, or evaluation of clinical efficacy, making the applicability of such applications unclear in the clinical setting.^13^,^34^,^49^,^52^,^53^,^57^,^58^,^61^,^63^,^65^ Evidently, the need for new methods to validate and evaluate such newly developed AI tools is essential.^73^ Although it is important to study the ability of a developed AI tool to accomplish its intended goal and validate its use in real clinical settings (i.e. ability to predict opioid requirements in the postoperative setting), these tools must ultimately be evaluated by their ability to have a clinically meaningful impact (i.e. ability to affect patient reported pain outcomes).^3^ Studies within this review that did evaluate their described intervention tended to evaluate the accuracy of their tool, rather than evaluating how their tool affects patient care. Ultimately, AI tools should be developed based upon clinical need, where the input of such tools would add value to the care that patients are receiving beyond that achievable without such systems. Therefore, future studies comparing the use of AI tools to clinical practice without such tools within similar clinical scenarios would be of value as few current studies accomplish this. Similarly, further research examining the advantage of described AI applications over currently used conventional clinical tools remains lacking. These investigations should ideally be performed in multiple healthcare contexts with diverse patient populations.

Studies described in this review also expressed concerns regarding a lack of provider comprehension and trust of the AI algorithms used within developed tools, which may hinder their use in the clinical setting.^38^^,^^39^ For such AI systems to ultimately improve patient outcomes, increased physician and healthcare worker education regarding such AI systems will be needed. It is important to note that applications of AI described in the literature did not intend to replace the role of perioperative clinicians, but rather to complement their abilities. Therefore, physicians must have a robust understanding of the AI tools that are used within their practice, as it is ultimately the physician who must appropriately apply and monitor such systems in the clinical setting. Improved provider understanding is also vital to improve user trust in such systems. As children are a particularly vulnerable population, particular attention will need to ensure that both clinicians and families have confidence in the clinical tools that are used to contribute to medical decision-making.^8^

Despite these ongoing challenges, the incorporation of AI technologies does hold the potential to offer new insights and greater accuracy of prediction using the large amount of data generated in the perioperative setting.^16^ The recent advances in AI within anaesthesia, particularly regarding machine learning, have been attributed to the combination of three factors: (1) the accessibility of large datasets, (2) the development of hardware able to perform intensive processing tasks, and (3) an uptake in development of AI techniques and algorithms.^4^ The application of AI in medicine is a promising area of development that is poised to be the future of modern healthcare.^3^ Care must be taken to ensure that future applications of AI in clinical practice are deployed in the right situation to answer an appropriate question or solve an applicable problem, while remaining conscious of the limitations of the given tool.^4^ Further work to improve the meaningful development, evaluation, and implementation of such tools remains active.^3^

Although this review adheres to previously published methodological frameworks for systematic reviews,^14^ this study has limitations. For instance, the heterogeneity of the included literature limited our ability to investigate each application of AI in detail, instead focusing on identifying and summarising trends in the literature. Similarly, the heterogeneity of the described applications limited our ability to compare the accuracy/efficacy of discussed AI tools as many were evaluated using distinct metrics. Finally, the scope of this review was limited owing to the English and French language restriction.

## Conclusions

There is emerging literature describing applications of AI for use within paediatric anaesthesia, and these technologies have the potential to advance patient care and ultimately improve patient outcomes. The literature suggests that current applications of AI are concentrated on patient risk factor prediction, anaesthetic depth estimation, anaesthetic medication/technique decision guidance, intubation assistance, airway device selection, physiological variable monitoring, and operating room scheduling. However, further work is necessary to address the multiple barriers that remain to the clinical integration of such technologies including lack of external validation and evaluation, tools based upon poor quality input data, and the unclear generalisability of applications to multiple patient populations and anaesthetic settings.

## Human rights

This article does not contain any studies with human participants performed by any of the authors.

## Authors’ contributions

Conceptualisation: RA, PI.

Data collection: RA, ES.

Data extraction: RA, ES.

Formal analysis: RA.

Search strategy development: GG.

Manuscript writing: RA.

Manuscript review and editing: ES, GG, PI.

Supervision: PI.
