# Supplementary material for: Use of artificial intelligence in paediatric anaesthesia: a systematic review
Source: BJA Open. 2023 Feb 7;5:100125. doi: 10.1016/j.bjao.2023.100125 (PMC10430814; doi:10.1016/j.bjao.2023.100125)
Supplement: Multimedia component 1 [file mmc1.docx]

**Appendix A – Search Strategy**

**Central**

Date: 07/05/2022 00:56:54

ID Search Hits

#1 (analgesi*:ti,ab,kw OR an?esthesi*:ti,ab,kw) OR perioperative:ti,ab,kw OR (Bupivacaine:ti,ab,kw OR Desflurane:ti,ab,kw OR Diazepam:ti,ab,kw OR Etomidate:ti,ab,kw OR Fentanyl:ti,ab,kw OR Isoflurane:ti,ab,kw OR Ketamine:ti,ab,kw OR Lidocaine:ti,ab,kw OR Midazolam:ti,ab,kw OR propofol:ti,ab,kw OR Sevoflurane:ti,ab,kw OR Sufentanil:ti,ab,kw OR Succinylcholine:ti,ab,kw OR Rocuronium:ti,ab,kw) 148623

#2 child*:ti,ab,kw OR paediatric*:ti,ab,kw OR paediatric*:ti,ab,kw OR prematur*:ti,ab,kw OR preterm*:ti,ab,kw OR perinat*:ti,ab,kw OR neonat*:ti,ab,kw OR ("neo" NEXT nat*):ti,ab,kw OR newborn*:ti,ab,kw OR ("new" NEXT born*):ti,ab,kw OR infan*:ti,ab,kw OR baby*:ti,ab,kw OR babies:ti,ab,kw OR toddler*:ti,ab,kw OR boy*:ti,ab,kw OR girl*:ti,ab,kw OR kid?:ti,ab,kw OR school*:ti,ab,kw OR juvenil*:ti,ab,kw OR underage*:ti,ab,kw OR ("under" NEXT age*):ti,ab,kw OR teen*:ti,ab,kw OR minor?:ti,ab,kw OR youth?:ti,ab,kw OR adolescen*:ti,ab,kw OR pubescen*:ti,ab,kw OR puberty:ti,ab,kw 356748

#3 ((artificial*:ti,ab,kw OR computat*:ti,ab,kw OR ensemble:ti,ab,kw OR machine*:ti,ab,kw OR multitask*:ti,ab,kw OR ("multi" NEXT task*):ti,ab,kw OR reinforcement:ti,ab,kw OR transfer:ti,ab,kw OR deep:ti,ab,kw OR supervi*:ti,ab,kw OR unsupervi*:ti,ab,kw OR shallow*:ti,ab,kw OR competitive:ti,ab,kw) NEAR/1 (intelligen*:ti,ab,kw OR learn*:ti,ab,kw)) 3330

#4 (computer*:ti,ab,kw NEAR/1 media*:ti,ab,kw NEAR/1 communicat*:ti,ab,kw) 13

#5 (natural-language:ti,ab,kw OR chat-bot?:ti,ab,kw OR chatbot?:ti,ab,kw OR (convers*:ti,ab,kw NEAR/2 (agent?:ti,ab,kw OR assistant?:ti,ab,kw))) 393

#6 ((bayes*:ti,ab,kw OR neural:ti,ab,kw OR deep:ti,ab,kw OR ("echo" NEXT state*):ti,ab,kw OR "generative adversarial":ti,ab,kw) NEAR/1 (network*:ti,ab,kw OR naive*:ti,ab,kw OR learning*:ti,ab,kw OR reservoir*:ti,ab,kw)) 2039

#7 ((automated:ti,ab,kw OR comput*:ti,ab,kw) NEAR/1 (heuristic:ti,ab,kw OR reasoning:ti,ab,kw OR soft:ti,ab,kw OR evolutionary:ti,ab,kw OR vision:ti,ab,kw)) 137

#8 ("data driven":ti,ab,kw OR ((data:ti,ab,kw OR text:ti,ab,kw) NEAR/1 mining:ti,ab,kw)) 613

#9 (fuzzy:ti,ab,kw NEAR/1 (logic:ti,ab,kw OR cluster*:ti,ab,kw OR cognit*:ti,ab,kw OR inference*:ti,ab,kw OR classific*:ti,ab,kw OR rule*:ti,ab,kw OR system*:ti,ab,kw OR control*:ti,ab,kw)) 90

#10 (knowledge*:ti,ab,kw NEAR/1 (acquisition*:ti,ab,kw OR representation*:ti,ab,kw)) 315

#11 ((adaptive:ti,ab,kw NEXT (boost*:ti,ab,kw OR system*:ti,ab,kw)) OR ((ambient:ti,ab,kw OR swarm:ti,ab,kw) NEXT intelligen*:ti,ab,kw) OR autoencoder*:ti,ab,kw OR ("auto" NEXT encod*):ti,ab,kw OR backpropagat*:ti,ab,kw OR ("back" NEXT propagat*):ti,ab,kw OR "dimensionality reduction":ti,ab,kw OR ("intelligent" NEXT control*):ti,ab,kw OR "k nearest":ti,ab,kw OR "learning to rank":ti,ab,kw OR metaheuristic*:ti,ab,kw OR ("meta" NEXT heuristic*):ti,ab,kw OR (predicti* NEXT model*):ti,ab,kw OR ("support" NEXT vector*):ti,ab,kw OR ((adaptive:ti,ab,kw OR expert:ti,ab,kw OR intelligent*:ti,ab,kw OR multiagent*:ti,ab,kw OR ("multi" NEXT agent*):ti,ab,kw) NEXT system*:ti,ab,kw) OR (markov:ti,ab,kw NEXT (chain*:ti,ab,kw OR model*:ti,ab,kw OR process*:ti,ab,kw))) 4543

#12 (random*:ti,ab,kw NEAR/2 forest*:ti,ab,kw) 599

#13 ((case-based:ti,ab,kw OR approximate*:ti,ab,kw OR automated:ti,ab,kw) NEAR/1 reasoning*:ti,ab,kw) 13

#14 ((bio-inspired:ti,ab,kw OR evolutionary:ti,ab,kw OR genetic:ti,ab,kw OR learning:ti,ab,kw OR clustering:ti,ab,kw) NEAR/1 algorithm*:ti,ab,kw) 634

#15 ((pattern*:ti,ab,kw OR document:ti,ab,kw) NEAR/1 classif*:ti,ab,kw) 64

#16 #3 OR #4 OR #5 OR #6 OR #7 OR #8 OR #9 OR #10 OR #11 OR #12 OR #13 OR #14 OR #15 9821

#17 #1 AND #2 AND #16 in Trials 42

**Embase Classic+Embase**

Date: <1947 to 2022 May 04>

1 exp artificial intelligence/ or big data/ or data mining/ or *software/ or computer interface/ or exp *machine learning/ or machine learning/ or semi supervised machine learning/ or supservised machine learning/ or unsupervised machine learning/ or exp *learning algorithm/ or natural language processing/ or expert system/ or fuzzy logic/ or fuzzy system/ or exp *robotics/ or *computer vision/ or *genetic algorithm/ or *computer prediction/ 284276

2 ((artificial* or computat* or ensemble or machine* or multitask* or multi task* or reinforcement or transfer or deep or supervi* or unsupervi* or shallow* or competitive) adj1 (intelligen* or learn*)).tw,kf. 129667

3 (computer* adj1 media* adj1 communicat*).tw,kf. 337

4 (natural-language or chat-bot? or chatbot? or (convers* adj2 (agent? or assistant?))).tw,kf. 9574

5 ((bayes* or neural or deep or echo state* or generative adversarial) adj1 (network* or naive* or learning* or reservoir*)).tw,kf. 118996

6 ((automated or comput*) adj1 (heuristic or reasoning or soft or evolutionary or vision)).tw,kf. 8100

7 (data driven or ((data or text) adj1 mining)).tw,kf. 36472

8 (fuzzy adj1 (logic or cluster* or cognit* or inference* or classific* or rule* or system* or control*)).tw,kf. 6657

9 (knowledge* adj1 (acquisition* or representation*)).tw,kf. 3925

10 ((adaptive adj (boost* or system*)) or ((ambient or swarm) adj intelligen*) or autoencoder* or auto encod* or backpropagat* or back propagat* or dimensionality reduction or intelligent control* or k nearest or learning to rank or metaheuristic* or meta heuristic* or predicti* model* or support vector* or ((adaptive or expert or intelligent* or multiagent* or multi agent*) adj system*) or (markov adj (chain* or model* or process*))).tw,kf. 149160

11 (random* adj2 forest*).tw,kf. 19892

12 ((case-based or approximate* or automated) adj1 reasoning*).tw,kf. 600

13 ((bio-inspired or evolutionary or genetic or learning or clustering) adj1 algorithm*).tw,kf. 36361

14 ((pattern* or document) adj1 classif*).tw,kf. 3099

15 or/1-14 505517

16 exp anesthesiological procedure/ 901335

17 anesthesiology/ 24568

18 exp *anesthetic equipment/ or anesthetic equipment/ 34134

19 exp anesthesist/ 38843

20 exp *anesthetic agent/ or anesthetic agent/ 302179

21 exp *analgesic agent/ or analgesic agent/ 493143

22 (analgesi* or an?esthesi*).mp,jw. 937833

23 perioperative.mp. 184255

24 (Bupivacaine or Desflurane or Diazepam or Etomidate or Fentanyl or Isoflurane or Ketamine or Lidocaine or Midazolam or propofol or Sevoflurane or Sufentanil or Succinylcholine or Rocuronium).mp. 381935

25 or/16-24 1989006

26 juvenile/ or exp adolescent/ or exp child/ or exp paediatrics/ 4250770

27 (child* or paediatric* or paediatric* or prematur* or preterm* or perinat* or neonat* or neo nat* or newborn* or new born* or infan* or baby* or babies or toddler* or boy* or girl* or kid$1 or school* or juvenil* or underage* or under age* or teen* or minor$1 or youth$1 or adolescen* or pubescen* or puberty).mp. 6040407

28 (neonat* or infan* or child* or adolescen* or paediatric* or paediatric*).jw. 968302

29 or/26-28 6153292

30 exp artificial intelligence/ or big data/ or data mining/ or *software/ or computer interface/ or exp *machine learning/ or machine learning/ or semi supervised machine learning/ or supservised machine learning/ or unsupervised machine learning/ or exp *learning algorithm/ or natural language processing/ or expert system/ or fuzzy logic/ or fuzzy system/ or exp *robotics/ or *computer vision/ or *genetic algorithm/ or *computer prediction/ 284276

31 ((artificial* or computat* or ensemble or machine* or multitask* or multi task* or reinforcement or transfer or deep or supervi* or unsupervi* or shallow* or competitive) adj1 (intelligen* or learn*)).tw,kf. 129667

32 (computer* adj1 media* adj1 communicat*).tw,kf. 337

33 (natural-language or chat-bot? or chatbot? or (convers* adj2 (agent? or assistant?))).tw,kf. 9574

34 ((bayes* or neural or deep or echo state* or generative adversarial) adj1 (network* or naive* or learning* or reservoir*)).tw,kf. 118996

35 ((automated or comput*) adj1 (heuristic or reasoning or soft or evolutionary or vision)).tw,kf. 8100

36 (data driven or ((data or text) adj1 mining)).tw,kf. 36472

37 (fuzzy adj1 (logic or cluster* or cognit* or inference* or classific* or rule* or system* or control*)).tw,kf. 6657

38 (knowledge* adj1 (acquisition* or representation*)).tw,kf. 3925

39 ((adaptive adj (boost* or system*)) or ((ambient or swarm) adj intelligen*) or autoencoder* or auto encod* or backpropagat* or back propagat* or dimensionality reduction or intelligent control* or k nearest or learning to rank or metaheuristic* or meta heuristic* or predicti* model* or support vector* or ((adaptive or expert or intelligent* or multiagent* or multi agent*) adj system*) or (markov adj (chain* or model* or process*))).tw,kf. 149160

40 (random* adj2 forest*).tw,kf. 19892

41 ((case-based or approximate* or automated) adj1 reasoning*).tw,kf. 600

42 ((bio-inspired or evolutionary or genetic or learning or clustering) adj1 algorithm*).tw,kf. 36361

43 ((pattern* or document) adj1 classif*).tw,kf. 3099

44 or/30-43 505517

45 25 and 29 and 44 1429

**Ovid MEDLINE(R)**

Date: 1946 to May 04, 2022

1 exp anaesthesia/ or anesthesiology/ [ADDED FIELD OF ANESTHESIOLOGY] 217231

2 "anaesthesia and analgesia"/ 3689

3 exp anesthetics/ or exp analgesics/ [ADDED EXP ANALGESICS] 753881

4 (analgesi* or an?esthesi*).mp,jw. [ADDED ANALGESICS AS KEYWORD AND JW TO SEARCH JOURNAL WORD FIELD] 621249

5 perioperative.mp. [ADDED] 122644

6 (Bupivacaine or Desflurane or Diazepam or Etomidate or Fentanyl or Isoflurane or Ketamine or Lidocaine or Midazolam or propofol or Sevoflurane or Sufentanil or Succinylcholine or Rocuronium).mp. [ADDED] 167111

7 or/1-6 1259906

8 exp infant/ or exp child/ or adolescent/ or exp paediatrics/ 3866267

9 (child* or paediatric* or paediatric* or prematur* or preterm* or perinat* or neonat* or neo nat* or newborn* or new born* or infan* or baby* or babies or toddler* or boy* or girl* or kid$1 or school* or juvenil* or underage* or under age* or teen* or minor$1 or youth$1 or adolescen* or pubescen* or puberty).mp. 5213973

10 (neonat* or infan* or child* or adolescen* or paediatric* or paediatric*).jw. 742883

11 or/8-10 5291958

12 exp artificial intelligence/ 145161

13 data mining/ 10169

14 big data/ 2180

15 *software/ 48447

16 exp user-computer interface/ 39066

17 ((artificial* or computat* or ensemble or machine* or multitask* or multi task* or reinforcement or transfer or deep or supervi* or unsupervi* or shallow* or competitive) adj1 (intelligen* or learn*)).tw,kf. 110169

18 (computer* adj1 media* adj1 communicat*).tw,kf. 293

19 (natural-language or chat-bot? or chatbot? or (convers* adj2 (agent? or assistant?))).tw,kf. 8213

20 ((bayes* or neural or deep or echo state* or generative adversarial) adj1 (network* or naive* or learning* or reservoir*)).tw,kf. 99178

21 ((automated or comput*) adj1 (heuristic or reasoning or soft or evolutionary or vision)).tw,kf. 7254

22 (data driven or ((data or text) adj1 mining)).tw,kf. 30238

23 (fuzzy adj1 (logic or cluster* or cognit* or inference* or classific* or rule* or system* or control*)).tw,kf. 5309

24 (knowledge* adj1 (acquisition* or representation*)).tw,kf. 3197

25 ((adaptive adj (boost* or system*)) or ((ambient or swarm) adj intelligen*) or autoencoder* or auto encod* or backpropagat* or back propagat* or dimensionality reduction or intelligent control* or k nearest or learning to rank or metaheuristic* or meta heuristic* or predicti* model* or support vector* or ((adaptive or expert or intelligent* or multiagent* or multi agent*) adj system*) or (markov adj (chain* or model* or process*))).tw,kf. 114424

26 (random* adj2 forest*).tw,kf. 15846

27 ((case-based or approximate* or automated) adj1 reasoning*).tw,kf. 484

28 ((bio-inspired or evolutionary or genetic or learning or clustering) adj1 algorithm*).tw,kf. 30155

29 ((pattern* or document) adj1 classif*).tw,kf. 2361

30 or/12-29 441859

31 7 and 11 and 30 833

**Scopus**

Date: May 6, 2022

(

TITLE-ABS-KEY(analgesi* OR an*esthesi* ) OR SRCTITLE(analgesi* OR an*esthesi* ) OR TITLE-ABS-KEY(perioperative) OR TITLE-ABS-KEY(Bupivacaine OR Desflurane OR Diazepam OR Etomidate OR Fentanyl OR Isoflurane OR Ketamine OR Lidocaine OR Midazolam OR propofol OR Sevoflurane OR Sufentanil OR Succinylcholine OR Rocuronium )

)

AND

(

TITLE-ABS-KEY(child* OR paediatric* OR paediatric* OR prematur* OR preterm* OR perinat* OR neonat* OR "neo nat*" OR newborn* OR "new born*" OR infan* OR baby* OR babies OR toddler* OR boy* OR girl* OR kid OR kids OR school* OR juvenil* OR underage* OR "under age*" OR teen* OR minors OR youth OR youths OR adolescen* OR pubescen* OR puberty ) OR SRCTITLE(neonat* OR infan* OR child* OR adolescen* OR paediatric* OR paediatric* )

)

AND

(

TITLE-ABS-KEY((artificial* OR computat* OR ensemble OR machine* OR multitask* OR "multi task*" OR reinforcement OR transfer OR deep OR supervi* OR unsupervi* OR shallow* OR competitive ) W/1 (intelligen* OR learn* )) OR TITLE-ABS-KEY(computer* W/1 media* W/1 communicat* ) OR TITLE-ABS-KEY(natural-language OR chat-bot* OR chatbot* OR (convers* W/2 (agent* OR assistant* ))) OR TITLE-ABS-KEY((bayes* OR neural OR deep OR "echo state*" OR "generative adversarial" ) W/1 (network* OR naive* OR learning* OR reservoir* )) OR TITLE-ABS-KEY((automated OR comput* ) W/1 (heuristic OR reasoning OR soft OR evolutionary OR vision )) OR TITLE-ABS-KEY("data driven" OR ((data OR text ) W/1 mining )) OR TITLE-ABS-KEY(fuzzy W/1 (logic OR cluster* OR cognit* OR inference* OR classific* OR rule* OR system* OR control* )) OR TITLE-ABS-KEY(knowledge* W/1 (acquisition* OR representation* )) OR TITLE-ABS-KEY((adaptive W/1 (boost* OR system* )) OR ((ambient OR swarm ) W/1 intelligen* ) OR autoencoder* OR "auto encod*" OR backpropagat* OR "back propagat*" OR "dimensionality reduction" OR "intelligent control*" OR "k nearest" OR "learning to rank" OR metaheuristic* OR "meta heuristic*" OR "predicti* model*" OR "support vector*" OR ((adaptive OR expert OR intelligent* OR multiagent* OR "multi agent*" ) W/1 system* ) OR (markov W/1 (chain* OR model* OR process* ))) OR TITLE-ABS-KEY(random* W/2 forest* ) OR TITLE-ABS-KEY((case-based OR approximate* OR automated ) W/1 reasoning* ) OR TITLE-ABS-KEY((bio-inspired OR evolutionary OR genetic OR learning OR clustering ) W/1 algorithm* ) OR TITLE-ABS-KEY((pattern* OR document ) W/1 classif* )

)

**Web of Science Core Collection (SCI-EXPANDED, CPCI-S, ESCI)**

May 6, 2022

291 results

(

TS=(analgesi* OR an$esthesi* ) OR TS=("perioperative") OR TS=(Bupivacaine OR Desflurane OR Diazepam OR Etomidate OR Fentanyl OR Isoflurane OR Ketamine OR Lidocaine OR Midazolam OR propofol OR Sevoflurane OR Sufentanil OR Succinylcholine OR Rocuronium )

)

AND

(

TS=(child* OR paediatric* OR paediatric* OR prematur* OR preterm* OR perinat* OR neonat* OR "neo nat*" OR newborn* OR "new born*" OR infan* OR baby* OR babies OR toddler* OR boy* OR girl* OR kid$ OR school* OR juvenil* OR underage* OR "under age*" OR teen* OR minor$ OR youth$ OR adolescen* OR pubescen* OR puberty )

)

AND

(

TS=((artificial* OR computat* OR ensemble OR machine* OR multitask* OR "multi task*" OR reinforcement OR transfer OR deep OR supervi* OR unsupervi* OR shallow* OR competitive ) NEAR/1 (intelligen* OR learn* )) OR TS=(computer* NEAR/1 media* NEAR/1 communicat* ) OR TS=(natural-language OR chat-bot$ OR chatbot$ OR (convers* NEAR/2 (agent$ OR assistant$ ))) OR TS=((bayes* OR neural OR deep OR "echo state*" OR "generative adversarial" ) NEAR/1 (network* OR naive* OR learning* OR reservoir* )) OR TS=((automated OR comput* ) NEAR/1 (heuristic OR reasoning OR soft OR evolutionary OR vision )) OR TS=("data driven" OR ((data OR text ) NEAR/1 mining )) OR TS=(fuzzy NEAR/1 (logic OR cluster* OR cognit* OR inference* OR classific* OR rule* OR system* OR control* )) OR TS=(knowledge* NEAR/1 (acquisition* OR representation* )) OR TS=((adaptive NEAR/0 (boost* OR system* )) OR ((ambient OR swarm ) NEAR/0 intelligen* ) OR autoencoder* OR "auto encod*" OR backpropagat* OR "back propagat*" OR "dimensionality reduction" OR "intelligent control*" OR "k nearest" OR "learning to rank" OR metaheuristic* OR "meta heuristic*" OR "predicti* model*" OR "support vector*" OR ((adaptive OR expert OR intelligent* OR multiagent* OR "multi agent*" ) NEAR/0 system* ) OR (markov NEAR/0 (chain* OR model* OR process* ))) OR TS=(random* NEAR/2 forest* ) OR TS=((case-based OR approximate* OR automated ) NEAR/1 reasoning* ) OR TS=((bio-inspired OR evolutionary OR genetic OR learning OR clustering ) NEAR/1 algorithm* ) OR TS=((pattern* OR document ) NEAR/1 classif* )

)
